# Seasonal Variations of Neuromotor Development By 14 Months of Age: Hamamatsu Birth Cohort for Mothers and Children (HBC Study)

**DOI:** 10.1371/journal.pone.0052057

**Published:** 2012-12-19

**Authors:** Kenji J. Tsuchiya, Hiroshi Tsutsumi, Kaori Matsumoto, Nori Takei, Makiko Narumiya, Maiko Honda, Ismail Thanseem, Ayyappan Anitha, Katsuaki Suzuki, Hideo Matsuzaki, Yasuhide Iwata, Kazuhiko Nakamura, Norio Mori, H. B. C. Study Team

**Affiliations:** 1 Research Center for Child Mental Development, Hamamatsu University School of Medicine, Hamamatsu, Japan; 2 United Graduate School of Child Development, Hamamatsu University School of Medicine, Hamamatsu, Japan; 3 Department of Psychiatry, Hamamatsu University School of Medicine, Hamamatsu, Japan; 4 Institute of Psychiatry, King’s College of London, London, United Kingdom; Chiba University Center for Forensic Mental Health, Japan

## Abstract

The present study aimed at investigating whether neuromotor development, from birth to 14 months of age, shows seasonal, cyclic patterns in association with months of birth. Study participants were 742 infants enrolled in the Hamamatsu Birth Cohort (HBC) Study and followed-up from birth to the 14th month of age. Gross motor skills were assessed at the ages of 6, 10, and 14 months, using Mullen Scales of Early Learning. The score at each assessment was regressed onto a trigonometric function of months of birth, with an adjustment for potential confounders. Gross motor scores at the 6th and 10th months showed significant 1-year-cycle variations, peaking among March- and April-born infants, and among February-born infants, respectively. Changes in gross motor scores between the 10th and 14th months also showed a cyclic variation, peaking among July- and August-born infants. Due to this complementary effect, gross motor scores at the 14th month did not show seasonality. Neuromotor development showed cyclic seasonality during the first year of life. The effects brought about by month of birth disappeared around 1 year of age, and warmer months seemed to accelerate the neuromotor development.

## Introduction

Seasonal fluctuations in birth rates have been observed in a wide range of populations around the world [1]. In the northern hemisphere, birth rates are usually high in the spring months [1]–[3], which has been thought to be related to higher conception rates in the summer to autumn months [2], [4]. In relation to this, patterns have also been documented in health and developmental outcomes of children in association with months of birth.

Studies have found that birthweight tends to be higher in the winter and spring months than in the summer and autumn months [5]–[9]. Similarly, winter-born children have been reported to grow fastest in terms of height during childhood [10], [11]. On the other hand, another study reported that the growth in height of infants speeds up during the summer months, irrespective of month of birth [12]. These findings suggest that seasonal patterns of developmental outcomes, if any, are connected with a complex picture of birth month seasonality, as well as seasonal effects on growth after birth.

Apart from anthropological measures, a limited number of studies have investigated neurodevelopmental outcomes in relation to seasonality of birth. McGrath et al. [13] reported that children born in winter and spring months (December to May) in a US cohort showed advanced motor skills at 8 months of age compared to those born in summer and autumn months. Similarly, Benson [14] found that age of locomotor onset (e.g., crawling) is earlier among those born during winter and spring months. In spite of this advantage among winter- and spring-born children, a recent study has shown that spring-born children are at higher risk of developing autism spectrum disorder (i.e., autism and related neurodevelopmental disorders) [15]. Furthermore, it has been well established that children born in winter and spring months are at higher risk of later diagnosis with schizophrenia [16]–[19], particularly in the northern hemisphere [20]. As a result, conflicting findings have emerged; some studies indicate that those born in winter or spring may have the advantage in neurodevelopment (especially neuromotor development) during the first year of life, whereas other studies suggest winter- and spring-born children are liable to impaired neurocognitive development in the long run.

Given these perplexing findings, the previously observed associations between neuromotor development in early life and seasonality of birth must be qualified from a number of viewpoints. First, there is a methodological limitation to the prior studies, since neuromotor development was compared between children born in winter and spring months and those born in other months [13], [14]; that is, seasonality was dichotomized or categorized. Any biological events related to seasons may be assumed to vary in a cyclic manner, and thus need to be handled with a proper statistical model that allows for a circular (trigonometric) function. Second, even if an advantage in neuromotor development among winter- and spring-born children is present at a very early stage of life, whether it lasts a long time or wanes and eventually disappears is not known. Third, the possibility remains that the neuromotor advantage, observed in previous studies, among winter- and spring-born children might be attributed to advanced development of motor functions that they achieve during some specific period of year *after birth* rather than to the season in which they were born.

In this study, we aimed to investigate whether neuromotor skills acquired at the age of 6, 10, and 14 months would display an invariable pattern that is expressed as a trigonometric function of seasonality of birth, using a representative birth cohort in Japan.

## Materials and Methods

This study was conducted as part of an ongoing cohort study (Hamamatsu Birth Cohort for Mothers and Children: HBC Study), which has been described in detail elsewhere [21].

### Participants

We consecutively contacted all the pregnant women (n = 926) who were expected to give singleton birth at either of our two research sites, the Hamamatsu University Hospital and Kato Maternity Clinic, both situated in Hamamatsu City, and who gave birth between 20 December, 2007 and 30 September, 2010. The enrolled parturients and their offspring were representative of Japanese parturients and their offspring in terms of age and socioeconomic status, parity, and birthweight and gestational age at birth [21]. All of the enrolled parturients were given a complete description of the study, and provided written informed consent to participate. The parturients were followed up from mid-pregnancy, when they were asked to enter into the study (between the 14th and 26th week of gestation), to 14 months after childbirth. Infants to whom the parturients gave birth were followed up from birth to 14 months of age.

After the follow-up, we excluded from analyses 186 participating infants (20%) either because they (N = 2) or their mother (N = 1) had died; because they had moved away (N = 10); because the parturient had moved to the city on a temporary basis for delivery and then moved away again, making it difficult for them to visit our research site during follow-up (N = 80); because the mother and the infant were lost to follow-up (N = 56); or because the infant was the child of a parturient who had already participated in the study after a previous birth (N = 37). The major reason for a temporary move among some parturients was “Satogaeri-bunben,” a traditional support system associated with childbirth in Japan, under which parturients move to their mother’s residence and are cared for by the mother during the perinatal period [22]. The reason for excluding the second children of a parturient who had already participated in the study after a previous birth was to render all the births independent events. The remaining 742 mothers and child dyads were entered into the analyses in this study.

We compared 184 excluded participants and 742 participants to be analyzed, and found that the parturient’s age and the age of the parturient’s partner were significantly younger in excluded participants than in the participants to be analyzed (mean age of the parturient = 30.5 vs 31.4 years, t(924) = 2.24, p = 0.03; mean age of the parturient’s partner = 32.3 vs 33.3 years, t(919) = 2.16, p = 0.03), although there were no significant differences between the two groups in terms of annual household income (mean = 5.5 vs 6.0 million JPY, t(924) = 1.94, p = 0.07), and gender (proportion of male infants = 54% vs 51%, χ^2^ (1) = 0.77, p = 0.38), birthweight (mean = 2,964 vs 2,971g, t(924) = 0.02, p = 0.83) and gestational age at birth (mean = 38.9 vs 39.0 weeks, t(924) = 0.82, p = 0.41).

During the follow-up period, five infants were found to suffer from conditions that are known to affect neuromotor development; 1 infant had a confirmed diagnosis of Down syndrome, 3 infants congenital heart defects with a range of severity, and 1 infant a hypothyroid functioning of unknown cause. As the number of these individuals was minimal and negligible compared with the total number of the participants, they were not eliminated from the analyses. However, as expected, the results remained unchanged after these cases were excluded (data not shown).

### Measurement

During the follow-up, the participating mothers and their infants were asked to visit our laboratory at the ages of 6, 10 and 14th months. To assess motor skills, we adopted the Mullen Scales of Early Learning (MSEL) [23]. MSEL is a composite scale for assessing child development through the age of 0 to 68 months, covering an area of development of gross motor skills. In MSEL, each infant’s gross motor skill is graded as an integer score ranging from 0 to 35 points, with an examiner starting from the first item to the ceiling item above which the infant fails to pass three consecutive items. Items for assessment were ordered along typical developmental trajectories, and we strictly followed the instructions.

Measurement of gross motor skills was performed in daytime by well-trained examiners who were kept blind to the data of previous assessments. The temperature of the observation rooms was set at a fixed temperature in the range of 21 to 27 degrees depending on the season.

Information on demographic and socioeconomic characteristics of mothers was collected during pregnancy of the enrolled parturients. These included ages of the mother and the partner (biological father of the offspring), parity, and annual household income (in million Japanese Yen; 1 million JPY = ca. 12,500 USD). Perinatal variables were collected from medical records, including gestational age and birthweight, as well as date and time of the birth. In addition to these, we examined whether the mother was breastfeeding the infant at the time of each examination of gross motor development: 6, 10, and 14 months of age.

In the present study, the 12 months of the year were separated into four seasons as follows: winter (December, January, February), spring (March, April, May), summer (June, July, August), autumn (September, October, November), according to the definition adopted in the related literature.

### Statistical Analysis

The assessment of gross motor scores was made within −30 to +30 days from the assigned date (the 6th, 10th, and 14th months of age). As such, there was sometimes a gap between the actual days of age and the recorded days of age at the time of assessment; there was no seasonal variation in the amount of this gap (data not shown). To adjust for this gap, we adopted a linear regression model to assess the relationship between age at the time of the assessment and gross motor scores. Then, we calculated the adjusted gross motor scores using the estimates from the linear regression model, which was built separately for the assessment at 6 months, 10 months, and 14 months of age. In the following analyses, we use these adjusted gross motor scores.

Before examining the seasonal variation of gross motor skills among infants in relation to month of birth, we considered the following variables as potentially exerting confounding effects. First, prematurity and birthweight were considered as variables that may have influenced the results when we examined the relationship between month of birth and neuromotor development and gender of the infant, as they have been suggested as such [24]–[26]. Then, parental ages, parity, annual household income as a socioeconomic indicator, and gender of the infant were also considered as candidates to be controlled for in the following analyses, since these variables may account for seasonal variation of births [27]. Furthermore, duration of breastfeeding is a major determinant of physical growth during infancy and childhood [11]. To test whether these variables should be entered into the following analyses because of potential confounding effects, we first checked whether these variables were statistically associated with seasons of birth, and then with gross motor scores at 6, 10, and 14 months of age. From a biostatistically conservative point of view, if any of these variables showed statistically significant associations (p<0.05) with either season of birth or gross motor scores at any one point of examination, by means of either t-test, chi-square test or linear correlation, they were regarded as potential confounders and thus were entered into the following analyses as fixed covariates to be adjusted (Table 1, 2).

**Table 1:** Characteristics of the participating infants [N = 742].

	Months of birth				
	Winter months (December, January, February)	Spring months(March, April, May)	Summer months(June, July, August)	Autumn months (September,October, November)	Statistics
Number of participants	161	188	207	186	χ^2^(3) = 2.92, p = 0.40
Gender of the infant					
Female	83 (52%)	93 (49%)	98 (47%)	91 (49%)	
Male	78 (48%)	95 (51%)	109 (53%)	95 (51%)	χ^2^ (3) = 0.65, p = 0.88
Parity					
First-born	82 (51%)	95 (51%)	124 (60%)	96 (52%)	
Second-born	52 (32%)	73 (39%)	66 (32%)	67 (36%)	
Third- and later born	27 (17%)	20 (11%)	17 (8%)	23 (12%)	χ^2^ (6) = 9.9, p = 0.13
Maternal age at birthin years (mean (SD))	32.1 (4.8)	31.0 (5.4)	31.1 (5.0)	31.4 (5.0)	F(3, 738) = 1.60,p = 0.19
Paternal age at birthin years (mean (SD))	34.4 (5.8)	32.9 (6.1)	32.9 (5.9)	33.4 (6.1)	F(3, 738) = 2.44,p = 0.06
Annual income of householdin million JPY (mean (SD))	6.3 (4.2)	6.1 (3.4)	5.7 (2.5)	5.9 (2.7)	F(3, 738) = 1.14,p = 0.33
Birthweight in gram (mean (SD))	2,985 (407)	2,981 (429)	2,948 (402)	2,971 (384)	F(3, 738) = 0.31,p = 0.81
Gestational age at birthin week (mean (SD))	39.1 (1.2)	39.2 (1.6)	39.0 (1.6)	39.0 (1.4)	F(3, 738) = 0.84,p = 0.47
Breastfeeding status					
Weaning completeby 6 months of age*	37 (23%)	48 (26%)	39 (19%)	53 (28%)	χ^2^ (3) = 5.43, p = 0.14
Weaning completeby 10 months of age*	63 (39%)	74 (39%)	73 (35%)	70 (38%)	χ^2^ (3) = 0.88, p = 0.83
Weaning complete by14 months of age*	99 (61%)	114 (61%)	123 (59%)	109 (59%)	χ^2^ (3) = 0.36, p = 0.95

* Cumulative number of children who have already been weaned from breastfeeding until each month of age.

**Table 2:** Mean gross motor scores at the 6th, 10th, and 14th months of age, and their statistical associations with potential confounders.

	6th month of age	10th month of age	14th month of age
Mean gross motor scores	7.4 (1.2)	12.6 (2.1)	18.0 (2.4)
Mean gross motor scores, stratified by gender of the child			
Female	7.4 (1.1)	12.7 (2.1)	17.9 (2.4)
Male	7.4 (1.3)	12.6 (2.1)	18.2 (2.5)
Test for equal means	F(1, 740) = 0.44, p = 0.51	F(1, 740) = 0.02, p = 0.90	F(1, 740) = 2.76, p = 0.10
Mean gross motor scores, stratified by parity			
First-born	7.4 (1.2)	12.6 (2.2)	18.0 (2.4)
Second-born	7.4 (1.3)	12.7 (1.9)	18.2 (2.5)
Third- and later born	7.4 (1.1)	12.6 (2.1)	17.7 (2.5)
Test for equal means	F(2, 739) = 0.11, p = 0.90	F(2, 739) = 0.44, p = 0.51	F(2, 739) = 1.47, p = 0.23
Correlation between mean gross motor scores and maternalage at birth: Correlation coefficients (r) and test for correlation	r = −0.04 p = 0.27	r = −0.10 p = 0.005	r = −0.07 p = 0.05
Correlation between mean gross motor scores and paternal ageat birth: Correlation coefficients (r) and testfor correlation	r = –0.01 p = 0.74	r = –0.05 p = 0.16	r = –0.06 p = 0.07
Correlation between mean gross motor scores and annualincome of household: Correlation coefficients (r) and test for correlation	r = 0.02 p = 0.53	r = –0.03 p = 0.38	r = –0.06 p = 0.10
Correlation between mean gross motor scores and birthweight:Correlation coefficients (r) and test for correlation	r = 0.15 p<0.001	r = 0.19 p<0.001	r = 0.19 p<0.001
Correlation between mean gross motor scores and gestationalage at birth: Correlation coefficients (r) and test for correlation	r = 0.15 p<0.001	r = 0.21 p<0.001	r = 0.14 p<0.001
Mean gross motor scores by breastfeeding status at6 months of age			
Weaning complete	7.4 (1.1)	Not measured	Not measured
Still breastfed	7.4 (1.4)	Not measured	Not measured
Test for equal means	F(1, 740) = 0.13, p = 0.72	Not tested	Not tested
Mean gross motor scores by breastfeeding status at10 months of age			
Weaning complete	Not measured	12.7 (2.1)	Not measured
Still breastfed	Not measured	12.5 (2.1)	Not measured
Test for equal means	Not tested	F(1, 740) = 0.41, p = 0.52	Not tested
Mean gross motor scores by breastfeeding status at 14 months of age			
Weaning complete	Not measured	Not measured	18.0 (2.5)
Still breastfed	Not measured	Not measured	18.0 (2.3)
Test for equal means	Not tested	Not tested	F(1, 740) = 0.01, p = 0.93

To assess fluctuations of adjusted gross motor function by month of birth at the ages of 6 months, 10 months, and 14 months, we adopted a linear regression model in which months of birth were transformed into a trigonometric form [16]. We regressed the mean value of adjusted gross motor scores in each of the 12 months onto a trigonometric function of months of birth and covariates. For simplicity, the predicted values of gross motor scores in each of the 12 months regressed onto a 0.5-year-cycle, 1-year-cycle, 2-year-cycle or 3-year-cycle trigonometric function of months of birth (*θ*) and its hypothetical phase (*θ*_0_), can be expressed in the following equation:
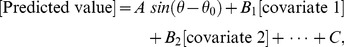
(1)where



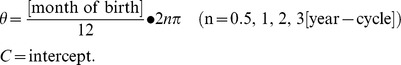



This was rearranged as 
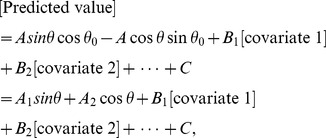
(2)where



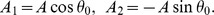



Using the linear regression model expressed in equation (2), we tested the null hypothesis of *A*_1_ =  *A*_2_ = 0, since it is appropriate to test *A*_1_ = 0 and *A*_2_ = 0 simultaneously as a pair to correspond to a periodic component of *θ* [28]. If the test statistics indicate a p-value <0.05, amplitude *A* and phase *θ*_0_ in the original equation (1) were determined as:
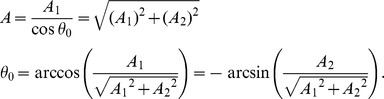



These estimates were shown with variance estimates, using the delta-method, as:
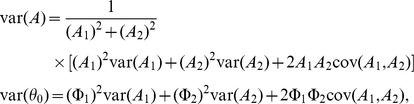
where



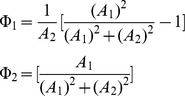



However, in the case that *A*_1_ =  *A*_2_ = 0 was not refuted, we judged the gross motor scores in each of the 12 months as showing no cyclic variation.

We preliminarily analyzed gross motor scores under the equation (2) with attention focused on which cycle best explained the models: a 0.5-year-, 1-year-, 2-year- or 3-year-cycle. As a result, no statistically significant association was found in gross motor scores at 6, 10, or 14 months of age regressed onto the 0.5-year-cycle, 2-year-cycle, or 3-year-cycle trigonometric function of month of birth. Thus, from this point onward, we only considered the periodicity of the trigonometric function as a 1-year cycle.

For statistical software, we used Stata version 12.1. All p-values were two-sided and statistical significance was set at 0.05.

### Ethical Issues

The study protocol was approved by the Hamamatsu University School of Medicine and the University Hospital Ethics Committee (no. 20–82, 21–114). Written informed consent to participate in this study, with allowance for withdrawal at any time from entry through follow-up, was obtained from each parturient, for her own participation and for her infant as the infant’s legal surrogate.

## Results

### Characteristics of Study Participants

Table 1 shows the characteristics of the 742 participating mother-and-infant dyads by seasons of birth of the infants. No marked or significant departure from expected numbers of births for the 4 seasons was evident (χ^2^(3) = 2.92, p = 0.40), indicating that seasons of birth of the participants were equally distributed. The gender of the infants, parental age at birth, parity, maternal age and paternal age at birth, annual household income, birthweight and gestational age at birth, and proportion of children kept breastfed at 6, 10, 14 months of age did not differ across seasons, either when using one-way ANOVA with Bonferroni correction or the chi-square test.

### Assessments of Gross Motor Development at 6, 10 and 14 Months of Age, and Testing of the Trigonometric Function for its Monthly Fluctuations

The first row in Table 2 shows mean scores of gross motor development at 6, 10, and 14 months of age. We also assessed whether gross motor scores were statistically associated with variables that may exert confounding effects, and found that maternal age at birth was significantly correlated with the score at the 10th and 14th months, and that birthweight and gestational age at birth were significantly correlated with the score at the 6th, 10th, and 14th months, as shown in Table 2. Whether the infant had been breastfed by the time of the neuromotor assessments was not associated with the gross motor scores. From this point onward, we determined that maternal age at birth, birthweight and gestational age at birth should be dealt with as potential confounders and thus entered in the following analyses.

Then, we tested models for gross motor scores at 6, 10, and 14 months of age regressed onto a 1-year cycle of trigonometric function of month of birth (upper 3 rows in Table 3), with an adjustment for three potential confounders. At 6 and 10 months of age, the trigonometrically transformed month of birth was significantly associated with gross motor development (F(2, 736) = 21.71, p<0.001 for 6 months, and F(2, 736) = 12.36, p<0.001 for 10 months of age). The predicted amplitude (A) and the phase (*θ*_0_) of the trigonometric function were 0.40 points and +0.52 months at 6 months, and 0.50 points and –0.82 months at 10 months of age. However, at 14 months of age, the 1-year cycle of trigonometric function of month of birth was not significantly associated with gross motor score (F(2, 736) = 1.21, p = 0.30). Therefore, the amplitude and the phase could not be predicted.

**Table 3:** Model statistics (R-squared, F-test and p-value), test statistics for an addition of trigonometric functions of months of birth (H_0_: A_1_ = A_2_ = 0), coefficients for the trigonometric functions, the amplitude and phase of trigonometric function of gross motor scores at 6, 10, and 14 months of age, and for differences between 6 and 10 months, and between 10 and 14 months of age.

	R-square, F-testand p-value	Test statistics for H_0_: A_1_ = A_2_ = 0	Coefficientfor A_1_	Coefficientfor A_2_	Amplitude	Phase in month
6th month of age	0.085 F(5, 736) = 11.72 p<0.001	F(2, 736) = 21.71, p<0.001	0.38 p<0.001	–0.11 p = 0.06	0.40 (0.39 to 0.41)	0.52 (0.50 to 0.54)
10th month of age	0.087 F(5, 736) = 11.54 p<0.001	F(2, 736) = 12.36, p<0.001	0.46 p<0.001	0.21 p = 0.06	0.50 (0.49 to 0.51)	–0.82 (–0.85 to –0.79)
14th month of age	0.045 F(5, 736) = 4.84 p<0.001	F(2, 736) = 1.21, p = 0.30	0.02 p = 0.86	–0.19 p = 0.12	NA	NA
Difference between6th to 10th months	0.038 F(5, 736) = 5.29 p<0.001	F(2, 736) = 5.27, p = 0.005	0.07 p = 0.47	0.32 p = 0.002	0.32 (0.31 to 0.33)	–2.57 (–2.62 to–2.52)
Difference between10th to 14th months	0.049 F(5, 736) = 7.15 p<0.001	F(2, 736) = 14.4, p<0.001	–0.44 p<.001	–0.41 p<.001	0.60 (0.57 to 0.63)	4.49 (4.45 to 4.54)

Footnotes for Table 3: Each model was adjusted for maternal age at birth, birthweight and gestational age at birth.

**Figure 1:**
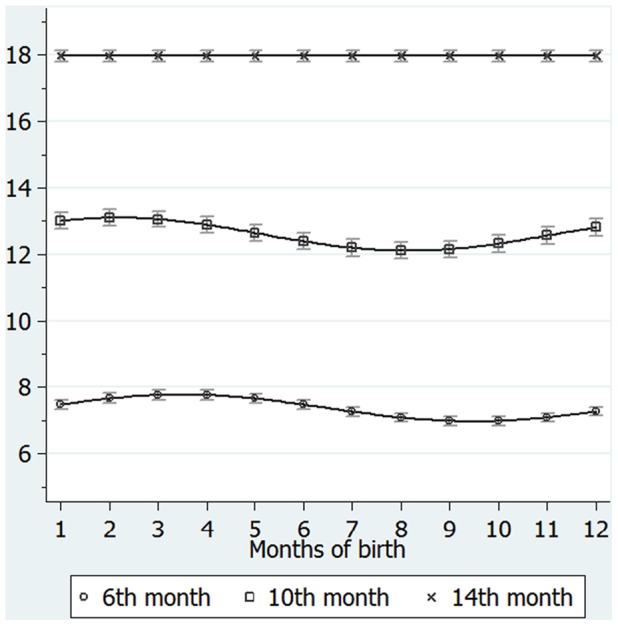
Gross motor scores at the 6th (circle), 10th (square), and 14th (X) months of life, as assessed by the Mullen Scales of Early Learning, predicted by trigonometric function of months of birth. Footnotes: Each curve and dot indicates predicted values of gross motor scores at the 6th, 10th, and 14th month regressed onto a trigonometric function of months of birth, adjusted for maternal age at birth, gestational age at birth and birthweight, with 95% confidence intervals. Statistics for trigonometric function, amplitude and phase in month at the 6th month assessment: F(2, 736) = 21.71, p<0.001, Amplitude = 0.40 (95%CI: 0.39 to 0.41) points, phase in month = 0.52 (95%CI: 0.50 to 0.54) months. At the 10th month assessment: F(2, 736) = 12.36, p<0.001, Amplitude = 0.50 (95%CI: 0.49 to 0.51), phase in month = –0.82 (95%CI: –0.85 to –0.79) months. At the 14th month assessment: F(2,736) = 1.21, p = 0.30, amplitude and phase were undetermined; predicted values are mean of gross motor scores adjusted for maternal age at birth, gestational age at birth and birthweight.

According to these results, Figure 1 shows predicted curves for gross motor scores with 95% confidence intervals at 6, 10, and 14 months of age, regressed onto the trigonometric function of month of birth, with an adjustment for maternal age at birth, birthweight and gestational age at birth. As can be seen from the Figure, the predicted gross motor score at 6 months of age peaked among March- and April-born infants, and was lowest among September- and October-born infants. The predicted gross motor score at the 10 months also showed significant monthly fluctuations, and peaked among February-born infants. The phase (*θ*_0_) of the sine function at 10 months of age was slightly shifted toward early months with a predicted value of –0.82 months.

### Difference in Gross Motor Scores between 6 and 10 Months of Age, and between 10 and 14 Months of Age

Early learners in gross motor skills at 6 months of age were more likely to be observed among those born in March and April, although this effect seemed to vanish beyond the first year of life, i.e., at 14 months of age. In order to reveal any changes in the pattern (velocity of development) of acquisition of gross motor skills through infancy at the individual level, we subtracted the gross motor score at 10 months of age from that at 6 months of age, and, similarly, the score at 14 from that at 10 months of age. Then, the model with a trigonometric function was again applied to these data.

Using this procedure, the test statistics and estimates for models for the subtracted difference in gross motor scores between 6 and 10 months and between 10 and 14 months of age regressed onto a 1-year-cycle trigonometric function of month of birth are shown in the lower 2 rows in Table 3. For the difference between 6 and 10 months of age, the trigonometrically transformed month of birth was significantly associated with gross motor development (F(2, 736) = 5.27, p<0.001). The predicted amplitude (A) and the phase (*θ*_0_) of the trigonometric function were 0.32 points and –2.57 months, respectively. For the difference between 10 and 14 months of age, the 1-year-cycle trigonometric function of month of birth was again significantly associated (F(2, 736) = 14.4), with the predicted amplitude (A) and the phase (*θ*_0_) of the trigonometric function being 0.60 points and +4.49 months, respectively.

**Figure 2:**
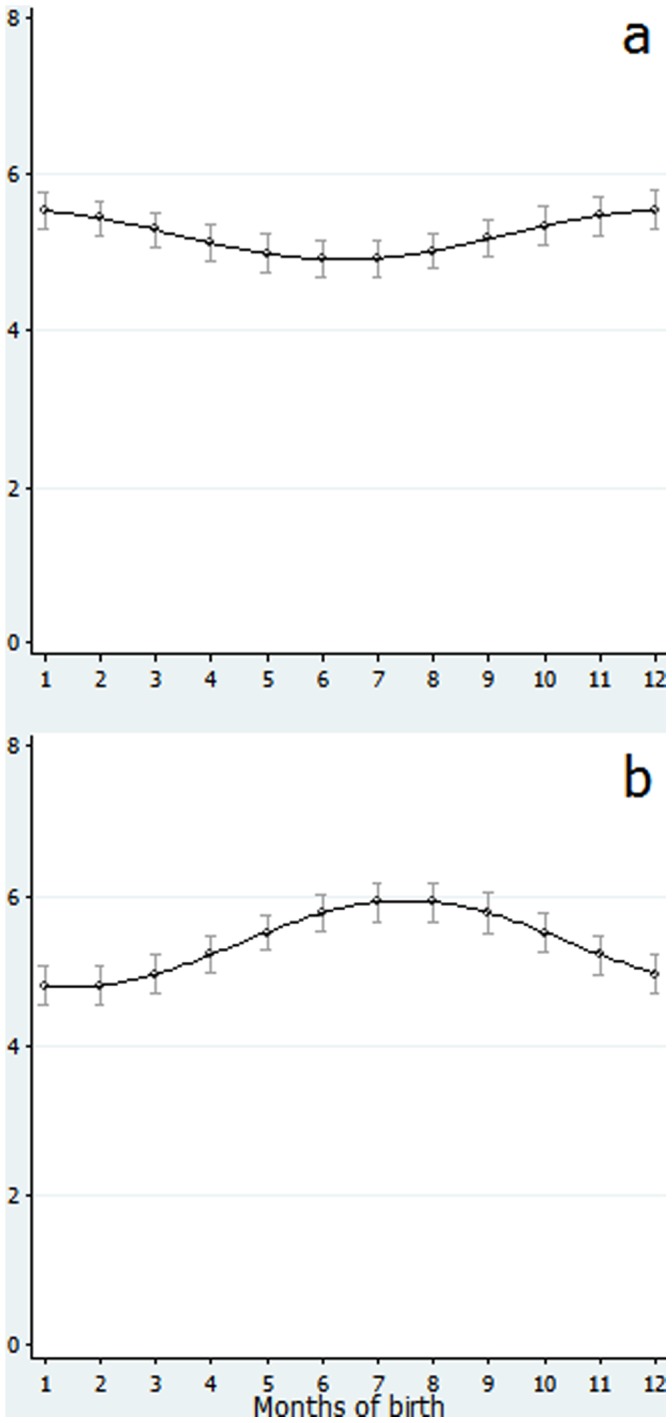
Difference in gross motor scores between (a) 6 and 10 months of age and between (b) 10 and 14 months of age. Each curve and dot indicates predicted values of difference in gross motor scores between (a) 6 and 10 months or (b) 10 and 14 months of age regressed onto a trigonometric function of months of birth, adjusted for maternal age at birth, gestational age at birth and birthweight, with 95% confidence intervals. Footnotes. (a) Statistics for trigonometric function, amplitude and phase in month in difference between 6 and 10 months: F(2, 736) = 5.27, p = 0.005, Amplitude = 0.32 (95%CI: 0.31 to 0.33) points, phase in month = –2.57 (95%CI: –2.62 to –2.52) months. (b) Statistics for trigonometric function, amplitude and phase in month in difference between 10 and 14 months: F(2, 736) = 14.4, p<0.001, Amplitude = 0.60 (95%CI: 0.57 to 0.63) points, phase in month = 4.49 (95%CI: 4.45 to 4.54) months.

Accordingly, we obtained two curves as shown in Figure 2a and 2b. Figure 2a shows the predicted values with 95% confidence intervals and curves for the difference in gross motor scores between 6 and 10 months of age, regressed onto a 1-year-cycle trigonometric function of month of birth, with an adjustment for maternal age at birth, birthweight and gestational age at birth. As can be seen from the Figure, the predicted difference in the score between 6 and 10 months peaked among December- and January-born infants. Similarly, Figure 2b shows the predicted difference in gross motor scores between 10 and 14 months of age, peaking among July- and August-born infants.

## Discussion

Using a representative sample of a birth cohort in Japan, we found a cyclic (1-year cycle) pattern of gross motor development, according to month of birth, at both 6 and 10 months of age. At 6 months of age, the peak of gross motor development was found among those born in spring months (March and April) and its trough was found among those born in autumn months (September, October). At 10 months of age, the cyclic pattern remains the same, with the exception of a slight (0.82 month) forward shift of its peak. However, this cyclic fluctuation of motor development according to month of birth disappears at 14 months of age, indicating that the advantage in neuromotor abilities acquired early in life (during the first year of life) by those born in winter and spring does not persist to 14 months of age.

In the analyses of subsequent changes in gross motor development between 6 and 10 months, and between 10 and 14 months of age by month of birth, a surprising pattern of development was revealed, especially for the latter period (i.e., 10 to 14 months of age). During this period, those born in summer and autumn achieved the highest performance in neuromotor skills, whereas this cyclicity is opposed to the pattern they had at an early stage of life (at 6 and at 10 months of age); in contrast, those born in winter and spring showed the lowest development. Consequently, the advantage of early-stage motor development conferred by spring birth was cancelled out by the later stagnant acquisition of motor skills at around one year of age (from 10 to 14 months), whereas the later-stage, catch-up acquisition of motor skills in the autumn-born infants compensated for their relatively delayed development early in life.

McGrath and colleagues [13] have reported that those born in winter/spring months have an advanced level of motor function at the age of 8 months when compared with those born in summer/autumn months. Likewise, Benson has demonstrated that infants born in winter/spring months commence crawling approximately 3 weeks earlier than infants born in other months. As these studies focused on the first year of life, our data in the first 10 months of age are mostly in line with their findings, although in the prior studies, the circular seasonality effect was not investigated. To our knowledge, the present study is the first to demonstrate an association between advanced motor skills and month of birth in a cyclic manner. In addition, the advantages of spring-born infants wane and disappear before the age of 14 months and, conversely, those born in autumn with relatively low levels of motor function at the early stage of development catch up and achieve the norm during the second year of life.

With respect to the possible explanations for these findings, biometeorological factors should be considered first. In this study, infants who showed the most advanced gross motor skills at 6 months of age were found to have been born in March and April, and thus had experienced the warmest months by the age of 6 months, while those born in September and October showed the least advanced gross motor development and had experienced the coldest 6 months after birth. Similarly, infants who showed the largest difference in gross motor skills between 6 and 10 months of age, namely, those born in December and January (see Figure 2a), were those who spent the warmest months during the corresponding period (e.g., December-born infants underwent their spurt in gross motor skill development during the months of June through September), while those who showed the largest changes in gross motor skills between 10 and 14 months of age, namely, those born in July and August (see Figure 2b), were again those who were in the warmest months during the period of improvement (e.g., July-born infants underwent their spurt in gross motor skill development during the months of May through August). Consistency was found in that those who showed the largest changes in gross motor scores were those who spent warmer months during the corresponding period. This is consistent with studies from China and Japan indicating that growth in body size is accelerated during warmer seasons [12], [29], [30], irrespective of month of birth. To this point, neuromotor advantages observed between the 6th and 10th months of age among spring-born children, and catch-up growth observed between the 10th and 14th months of age among summer/autumn-born children, are likely attributable not to birth month per se, but rather to the season in which the spurt in gross motor skill actually occurs.

Before providing further interpretations of these observations, therefore, we must look at climate conditions in Hamamatsu, which shows substantial seasonal variations in ambient temperature (Table 4); the minimum monthly average temperature of 5.9 degrees Celsius occurs in January, and the maximum 27.0 degrees Celsius occurs in August. The months with a monthly average temperature over 15.0 degrees are May, June, July, August, September and October [31], indicating that Hamamatsu, where our research was conducted, has a warm mild climate with six months of warm months. Considering the ambient temperature pattern in Hamamatsu, neuromotor development in the infants appears to have been accelerated during the warmer months in a dose-response fashion.

**Table 4:** Ambient temperature, precipitation, and duration of sunlight in Hamamatsu, Japan (34^o^42.5′N 137^o^43.1′E: 1981–2010). [31].

	Monthly mean temperature (degrees)	Monthly mean daily maximum temperature (degrees)	Monthly mean daily minimum temperature (degrees)	Monthly total ofsunlight duration(hours)	Monthly total of precipitation (mm)
January	5.9	10.1	2.5	196.5	57.0
February	6.5	11.1	2.7	184.2	78.3
March	9.7	14.3	5.6	191.0	149.4
April	14.7	19.3	10.4	195.6	167.5
May	18.7	23.0	14.9	195.8	190.5
June	22.0	25.8	19.0	148.3	241.3
July	25.7	29.4	23.0	177.5	190.0
August	27.0	31.1	24.0	222.6	150.8
September	24.1	28.2	21.0	161.0	248.9
October	18.8	23.1	15.3	165.9	164.5
November	13.5	17.9	9.8	170.0	118.8
December	8.4	12.7	4.8	199.5	52.3
Yearly	16.3	20.5	12.8	2207.9	1809.1

Even under the assumption that all or some of the advance or delay in neuromotor development at an early stage of life was attributable to exposure to climate conditions over the period of early development, however, the link between ambient temperature and neuromotor development is not straightforward. This is because biometeorological changes across seasons may be linked with a highly intermingled matrix of exposures, e.g., physical activities, clothing, availability of food, nutritional intake, metabolic and endocrinological status, and photoperiodicity [13], [14], [32]–[34]. All of these elements may be relevant to our findings.

First, seasonality patterns in physical activity among children have been widely supported by the literature, with physical activity being high in the warm months and low in cold months [35], [36], and thus enhanced physical activity during the warm months could be connected with advantages in gross motor development. However, seasonal fluctuation in physical activity has been reported to show a regional difference [36]; that is, children in Canada, a country with long, cold winter months, were reported to be hindered from outdoor activity during winter [37], [38]. On the other hand, another study conducted under a warmer climate did not support the existence of such seasonal fluctuation [39], and a study performed in Galveston, Texas actually showed decreased physical activity among infants during the summer months because of heat [40]. Considering that the average summer maximum temperature in Hamamatsu is as high as that in Galveston, Texas, it might be expected that the children in our study did not show enhanced physical activity in summer. In addition, the average winter temperature is not very low in Hamamatsu, and thus any decrease in physical activity due to winter weather would not be as pronounced as in countries like Canada. Given the recent progress in standard of living in the area where this study was conducted, which has allowed for widespread adoption of heating (above 99%) and air-conditioning (95%), the indoor and outdoor physical activity levels in our sample during cold months and warm months can be assumed to be neither highly enhanced nor dramatically decreased.

In relation to this, we have noted that the results of gross motor examination are affected by the ambient temperature of our examination rooms, and to how heavily (or lightly) the child is dressed, since children wearing coats, sweaters, or other extra layers of clothing will naturally be limited in their physical activity and thus their gross motor scores will be affected. We therefore set the room temperature at between 21 and 27 degrees depending on season, allow the child to dress as minimally as possible without feeling hot or cold, and allow the child to become accustomed to the room temperature and conditions for a sufficient amount of time before beginning the examinations.

Second, seasonal changes in availability of food and resultant nutritional intake may affect not only physical growth but also various domains of development in children. In developing countries, there have been observations indicating that season, including seasonal pattern of rain fall, highly influence the availability of food and nutritional patterns among pre-school-aged children [41], [42]. Furthermore, the costs for agricultural products may vary largely across seasons. However, this is not the case in highly modernized countries such as US and Japan, where the level of retailer competition is high, since pricing and availability for most fruits and vegetables is stable [43]. Studies have reported that energy intake has a seasonal fluctuation both in young children and in adults in Western countries, but the magnitude of difference was limited [44], [45]. Furthermore, whether the participating infants were breastfed at the time of the examinations did not influence the gross motor scores (Table 2). Therefore, we assumed that food availability did not play an important role in explaining the seasonal variation of gross motor development in our study.

As for another seasonal aspect of nutrition, Vitamin D has been shown to play a critical role in infants for the development of bone mass [11] as well as brain and cognitive development [46], [47], the level of which has been known to show seasonality both in pregnant women and infants [48], [49] with higher levels in sunny months [50]. For newborns, the only source of vitamin D is a maternal one via placenta, thus exclusively breastfed infants are likely to suffer from subclinical vitamin D deficiency [50], [51]. One study from Japan reported that the prevalence of vitamin D deficiency shows seasonality, with the highest prevalence of approximately 30% was found among April-, May- and June-born Japanese infants at the 1st month of age [51]. Apparently, this is inconsistent with our findings indicating spring-born children, who are expected to reveal a high level of vitamin D [52], showed an accelerated gross motor development by the 10th month of age. To account for this inconsistency, it is possible that the infants born in spring months in this study might be more likely to be fed with formula with a higher level of vitamin D. Nevertheless, we did not find any association between breastfeeding status and months of birth (Table 1), nor between breastfeeding status and the gross motor development score Table 2). Thus far, we could not find any supports for relevance of vitamin D to seasonal variations of neuromotor development, although direct investigations on maternal as well as infants’ level of vitamin D and of sunlight exposure are required.

Technical explanations for our findings of seasonal fluctuation of gross motor development in infancy include seasonal variation in birthweight and gestational age at birth, as some studies suggest that neonates born in winter and spring months have lower birthweight and shorter gestational age at birth compared with those born in other months [5], [8], [30], [53]. We did not find such a variation in our sample, as shown in Table 1. In fact, when we entered birthweight and gestational age at birth into the regression models as covariates, seasonal variation of gross motor function remained highly significant.

In relation to this, body weight at the age of 6, 10, and 14 months may account for seasonal fluctuation of gross motor development, as body weight increase has been indicated to be accelerated during summer months during the first and second years of life [7], [12]. Unfortunately, we did not measure body weight at the 6th and 14th month. However, the available data at 10 months of age indicates that there was no significant difference in mean body weight in the 10th month (winter-born 8,565g, spring-born 8,596g, summer-born 8,622g, autumn-born 8,468g: F(3, 739) = 1.11, p = 0.34). When body weight at 10 months of age was entered into the regression model, in which gross motor development was regressed onto the trigonometric function of months of birth with an adjustment for maternal age at birth, gestational age at birth and birthweight, the trigonometric function remained statistically significant.

In conclusion, we observed a variability of neuromotor development according to season of birth (i.e., an advantage conferred by birth in the spring months and a delay observed in infants born in the autumn months) by 10 months of age, and we found that these cyclically patterned seasonal fluctuations observed in early infancy had completely disappeared at around one year of age. This is because the delayed motor development in the autumn-born infants was cancelled out by a catch-up period at from 10 to 14 months of age. With respect to the mechanism underlying these findings, the ambient temperature to which infants are exposed in the early stage of life may account for some, but not all, of the differing patterns of motor development. As we continue to clarify the overall phenomenon of variations in neuromotor development according to birth season, elucidation of the “catch-up” phenomenon in particular may help to advance our understanding of developmental biology and chronobiology.

### Limitations

Our findings should be interpreted in the context of the following methodological limitations. First, the sample size in this study was relatively small. Especially when seasonal patterns are evaluated by merely examining fluctuations (deviations) in the monthly number of births, a large sample size is required. However, our interest in this study was to detect differences in the mean scores of gross motor skills across different birth months. We confirmed that, in a power calculation analysis, the numbers of participants assigned to each month of birth provided a sufficient power (>80%) to detect a significant (alpha = 0.05) difference. Furthermore, a cyclic trend analysis applied in this study involved estimation of fewer numbers of parameters than ordinary analyses used to examine seasonality considering the effects of each of the separate 12 months; that is, the parsimonious models chosen in this study provided additional statistical power. Second, we focused on gross motor but not on fine motor performance, because the former is regarded as the dominant domain of neurodevelopment, particularly during the first year of life [23]. However, other domains including language development during the first or second year of life should be investigated in the forthcoming studies. In relation to this, we relied on a sample of the birth cohort study which is an ongoing research project. Detailed assessments of subsequent development in various domains, including fine motor functions and cognitive acquisition of skills including language and social skills are under way. At this stage, we are unable to ascertain any influences of advantage (or delay) in neuromotor development at the early stage of life on other domains over the subsequent course of the development. Third, we did not investigate physical health of the participating infants. For instance, infants are expected to have infectious diseases more likely during winter months than during other months; seasonal variations in compromised physical health, even if the morbid period is short, may account for the seasonal variations of gross motor development.
