# Correction: Seasonal Variations of Neuromotor Development By 14 Months of Age: Hamamatsu Birth Cohort for Mothers and Children (HBC Study)

**DOI:** 10.1371/annotation/ce119959-9626-4a3e-a30f-5b9f1bb40a2d

**Published:** 2013-08-06

**Authors:** Kenji J. Tsuchiya, Hiroshi Tsutsumi, Kaori Matsumoto, Nori Takei, Makiko Narumiya, Maiko Honda, Ismail Thanseem, Ayyappan Anitha, Katsuaki Suzuki, Hideo Matsuzaki, Yasuhide Iwata, Kazuhiko Nakamura, Norio Mori, H. B. C. Study Team

The Acknowledgments section is missing author and affiliation information about the H. B. C. Study Team. The following is the list of authors and their affiliations:

The HBC study team includes: Ms. Riyo Takabayashi 1, Yukiko Suzuki 1, Yumeno Kugizaki 1, Hiroko Muraki 1, Yui Seno 1, Emiko Kawai 1,2, Noriko Kodera 1, Emi Higashimoto 1, Atsuko Nakamura 1, Eriko Sato 1, Drs. Naohiro Kanayama 3, Hiroaki Itoh 3, Chie Shimmura 1, Yujin K. Kuroda 1, Shun Takahashi 1,2, Tsuruko Mori 1,2, Keiko Iwata 1,2, Yosuke Kameno 1,4, Yujiro Yoshihara 1,4, Shu Takagai 4, Tomoyasu Wakuda 4, Kiyokazu Takebayashi 4, Daisuke Kurita 4, Masayoshi Kawai 4, Shiro Suda 1, Genichi Sugihara 1, Kyoko Nakaizumi 1, Koichi Hirano 5, Yusaku Endoh 5, Teruhiko Suzuki 5, Toshirou Sugiyama 2,6, and Masatsugu Tsujii 1,2,7.

1 Research Center for Child Mental Development, Hamamatsu University School of Medicine, Hamamatsu, Japan,

2 United Graduate School of Child Development, Hamamatsu University School of Medicine, Hamamatsu, Japan,

3 Department of Obstetrics and Gynaecology, Hamamatsu University School of Medicine, Hamamatsu, Japan,

4 Department of Psychiatry, Hamamatsu University School of Medicine, Hamamatsu, Japan,

5 Department of Pediatrics, Hamamatsu University School of Medicine, Hamamatsu, Japan,

6 Department of Child Psychiatry, Hamamatsu University School of Medicine, Hamamatsu, Japan,

7 Faculty of Modern Sociology, Chukyo University, Nagoya, Japan

